# Bayesian spatio-temporal analysis of the COVID-19 pandemic in Catalonia

**DOI:** 10.1038/s41598-024-53527-w

**Published:** 2024-02-20

**Authors:** Pau Satorra, Cristian Tebé

**Affiliations:** Biostatistics Support and Research Unit, Germans Trias i Pujol Research Institute and Hospital (IGTP), Badalona, Barcelona Spain

**Keywords:** Statistics, Epidemiology

## Abstract

In this study, we modelled the incidence of COVID-19 cases and hospitalisations by basic health areas (ABS) in Catalonia. Spatial, temporal and spatio-temporal incidence trends were described using estimation methods that allow to borrow strength from neighbouring areas and time points. Specifically, we used Bayesian hierarchical spatio-temporal models estimated with Integrated Nested Laplace Approximation (INLA). An exploratory analysis was conducted to identify potential ABS factors associated with the incidence of cases and hospitalisations. High heterogeneity in cases and hospitalisation incidence was found between ABS and along the waves of the pandemic. Urban areas were found to have a higher incidence of COVID-19 cases and hospitalisations than rural areas, while socio-economic deprivation of the area was associated with a higher incidence of hospitalisations. In addition, full vaccination coverage in each ABS showed a protective effect on the risk of COVID-19 cases and hospitalisations.

## Introduction

The COVID-19 pandemic posed an unprecedented challenge to public health systems around the world and motivated the need for comprehensive epidemiological measures to monitor and respond effectively to the outbreak. In Spain, COVID-19 began to spread in March 2020. By July 2022, in which data were no longer available in open access, more than 2.6 million cases of COVID-19 were reported in Catalonia with 118,000 hospitalisations and 28,000 deaths, distributed in six registered different waves^1^. The geographical distribution of the spread of the pandemic was not spatially homogeneous in the Catalan territory and important differences were observed at the level of small areas, called basic health areas (ABS)^2^. The analysis of small units of space and time, where decisions often need to be made, is generally characterised by presenting high variability and noise, and traditional approaches may struggle to provide accurate estimates^3^. Using spatial and spatio-temporal disease mapping models, we can overcome many of these challenges by borrowing strength from spatial and temporal neighbours, allowing us to obtain reliable estimates for these small units and to uncover and understand the patterns of disease spread across space and time^4^.

There have been several studies worldwide analysing the spatial and spatio-temporal dynamics of COVID-19. These studies have used a wide variety of spatial and spatio-temporal methods, which are described in a systematic review^5^. 85% of the studies used frequentist approaches, while only 15% used a Bayesian approach. However, Bayesian methods are often preferred to frequentist methods because they allow a large number of components to be included using a hierarchical modelling scheme, which allows them to identify spatio-temporal patterns and hotspots^6^, or to also assess the impact of some explanatory variables^7–10^. Among many different other factors, these previous studies have seen how areas with a low socio-economic status and higher population density were associated with a higher risk of COVID-19 infections.

Since the development and introduction of COVID-19 vaccines, extensive work has been carried out to evaluate the vaccine’s efficacy and effectiveness and show its protective effect against COVID-19, particularly against severe disease^11–14^. Fewer studies have examined the role of COVID-19 vaccination at the local level using spatio-temporal models^15,16^. These studies find a significant impact of vaccination in the role of containing COVID-19 incidence at the local level.

The objective of this study was to investigate the spatio-temporal evolution of the incidence of reported COVID-19 cases and hospitalisations in the different ABS of Catalonia during the pandemic period. The effect of ABS demographic and socio-economic factors on COVID-19 cases and hospitalisations was also assessed, along with the effect of the percentage of vaccinated population.

## Methods

### Study design

An ecological study of administrative data was carried out to analyse the incidence of COVID-19 cases and hospitalisations, and the contextual factors that characterise ABS in Catalonia.

### Population and setting

**Figure 1:**
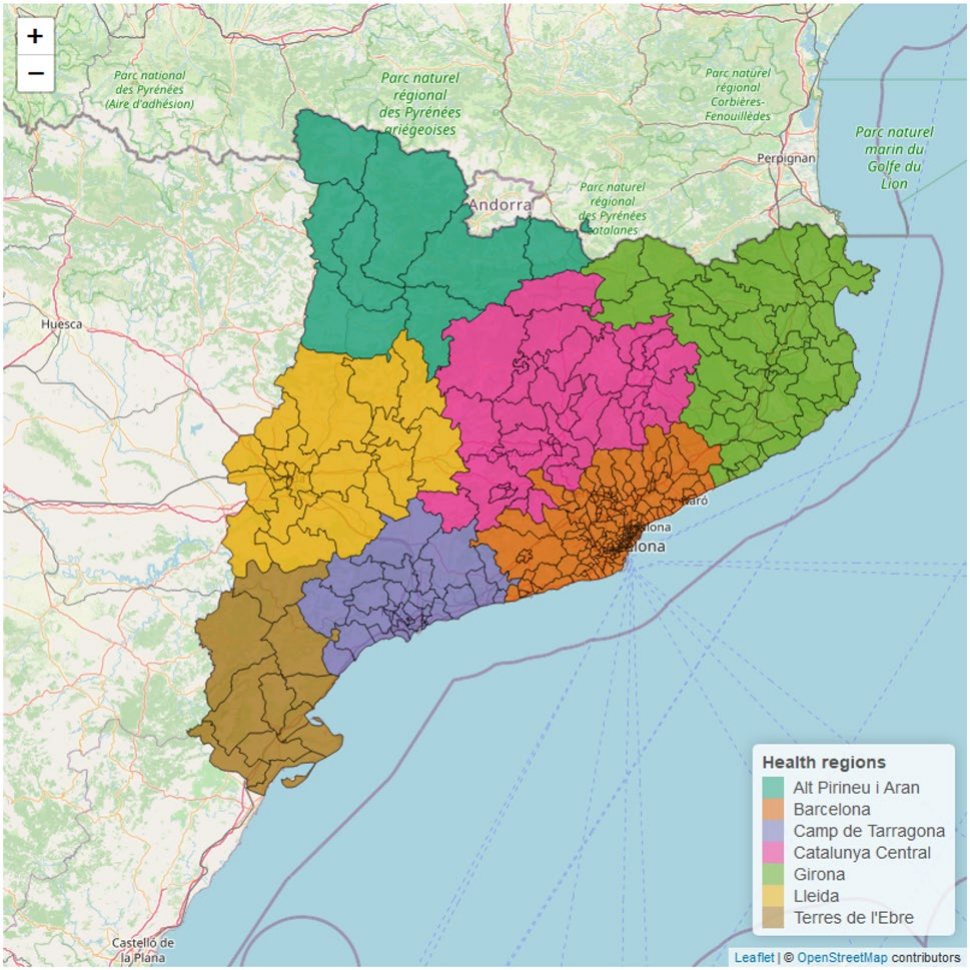
Map of the study territory, Catalonia. The territory is divided into 373 basic health areas (ABS), which are grouped into 7 different health regions, each represented by a different colour. Figure generated in R version 4.3.0.

Catalonia (32,113 km^2^) is a region in northeastern Spain with 7.6 million inhabitants living in 947 different municipalities grouped into seven different health regions. These regions are composed of 373 basic geographical units through which primary health care service is coordinated, called basic health areas (ABS). These small areas are defined by geographical, demographic, social and epidemiological factors, with the aim of guaranteeing equitable health resources and population’s accessibility to services. The map of Catalonia divided by ABS and health regions is shown in Fig. 1.

The study period started on 2020-03-01, the earliest date for which cases were available, and ended on 2022-07-24, the last week for which cases and vaccination data were available, comprising a total of 125 epidemiological weeks. Based on the inherent cyclical nature of the disease, we divided this time into different periods or waves. Although there is no standard definition of a wave^17^, the Catalan and Spanish health authorities identified six different waves throughout all the study time. We confirmed these waves in our data according to the peaks and valleys of the weekly reported COVID-19 incidence rate.

### Study data

All data came from the official open data catalogue of the Government of Catalonia, which is publicly available online^18^. Data on COVID-19 cases were available by day, health region, ABS, sex and age group from the epidemiological surveillance services^19,20^. Data on COVID-19 hospitalisations were available by week, ABS, sex and age group^21^. Data on COVID-19 vaccination were available by day and ABS^22^. The ABS socio-economic index was obtained from the Catalan Health System Observatory (OSSC)^23^. This index is a deprivation score, where the higher the score, the more deprived the area, and is made up of different socio-economic indicators standardised by age and aggregated by weights. These indicators are: population exempted from pharmaceutical co-payment, population with income < 18,000 euros, population with income > 100,000 euros, population with manual employment, population with inadequate level of education, premature mortality and avoidable hospitalisations. An urban-rural indicator was defined in function of the population density of the ABS and the number of ABS within the same city. More details on these indicators can be found in Supplementary Table S1. The geographical distribution of the urban-rural indicator and the socio-economic index by ABS is shown in Supplementary Fig. S1.

### Data processing

#### COVID-19 cases and hospitalisations

Daily data for COVID-19 cases were aggregated by each of the 125 epidemiological weeks, from 2020-03-01 to 2022-07-24, while hospitalisation data were directly reported on a weekly basis. We excluded those cases and hospitalisations for which it was not possible to identify the ABS of residence.

To measure the lack or excess of disease risk in an area, the age and sex standardised incidence ratio (SIR) was estimated^24^:

$$\begin{aligned} \text {SIR}_{it} = Y_{it}/E_i \end{aligned}$$

where $Y_{it}$ is the observed number of cases/hospitalisations in the *i*-th ABS and $E_i$ is the expected number that the area would have if it behaved like the general population of all Catalonia in average for the whole period. The expected counts were calculated using indirect standardization, knowing the age and sex distribution of cases/hospitalisations in the general population:

1 $$\begin{aligned} E_i = \sum \limits _{j=1}^ \text{J} r_jN_j \end{aligned}$$

being $N_j$ the population in the *j*-th sex-age stratum of the specific area and $r_j$ the average rate for the whole period in the same stratum:

$$\begin{aligned} r_j = \frac{\sum \nolimits _{i=1}^{n}\sum \nolimits _{t=1}^{T}Y_{itj}}{\sum \nolimits _{i=1}^{n}\sum \nolimits _{t=1}^{T}N_{itj}} \end{aligned}$$

The $\text {SIR}_{it}$ represents whether the area in a given time point has a higher ($\text {SIR}_{it} > 1$) or lower ($\text {SIR}_{it} < 1$) risk than would be expected from the general population on average in the whole period. For the vaccination analysis, reference rates $r_j$ were instead calculated independently for each time point *t* resulting in different $E_{it}$ for different time points. The $\text {SIR}_{it}$ calculated in this way represents the lack/excess of risk of a given area at a given time point compared to the general population at the same time point. This was used as a strategy to reduce the potential confounding effect of time, as explained later.

#### Vaccination

Daily data for COVID-19 vaccination was aggregated by week. We also excluded cases where it was not possible to identify the ABS of residence of the person to whom the dose was administered. The vaccination data varied not only in space, like the previous covariates, but also in time. It is well known that the analysis of time-varying covariates in observational studies is a challenging task and some additional analytical issues needed to be considered^25^.

First, it was necessary to define the time lag relationship between exposure and outcome. Vaccine effectiveness has been widely reported in the literature to occur 7 days after the second dose^11–13^. Therefore, we only considered complete vaccination as administered second doses of any vaccine or single doses of the one-shot vaccine and assumed that the effect occurs, at least, in the following week. Furthermore, studies have shown that the efficacy of the COVID-19 vaccine remains high time after full vaccination, although it decreases by 6 months, more so against symptomatic infections than against severe COVID-19 disease^14^. Consequently, vaccines administered in one week may have an effect from the following week onwards, so we used cumulative vaccination data to consider the entire vaccination history up to the previous week.

Another fundamental problem with studying a time-varying exposure effect in an observational study is the fact that there may be unobserved time-dependent confounders that affect both the exposure and the outcome. The conditions of the pandemic changed as it progressed, a larger infected population meant more people immunised, more clinical and social knowledge about the virus, different levels of policy and availability of testing, different restrictions in place, and even different COVID-19 variants, which may simultaneously affect the exposure variable and the study outcome^16^. To address this potential confounding effect, the expected counts were first calculated independently for each week, so that the SIR represents the risk of an area compared to the whole territory in the same week, and it no longer depended on the time trend of the outcome. Second, the analysis was stratified by waves in which the potential confounding conditions should be more similar, in order to try to remove any bias that might have arisen from differences in these conditions^26^. To do this, we considered separately the third and fourth waves (start of the vaccination campaign) and the fifth wave (dominated by the delta variant). The sixth wave was excluded from this analysis because, by this time, the majority of the population of each ABS had already received the second dose, as full vaccination coverage reached values above 80-90% in all ABS in Catalonia. Therefore, we couldn’t expect any effect due to such a small proportion of the population being fully vaccinated during this period. Moreover, only individuals aged 70 and over were included in the analysis of the third and fourth waves, as it was mainly the oldest group of people who were fully vaccinated at the beginning of the campaign. Finally, only hospitalisations were analysed in this period as we do not have information on reported COVID-19 cases by age group at the ABS level.

For this analysis, the study period started one week after we have enough fully vaccinated individuals to account for the week lag in vaccination. In particular, the first week with more than 5000 fully vaccinated individuals was the week starting at 2021-01-18, so we started one week later, at 2021-01-25, to account for the one week lag. The study period ended with the end of the fifth wave, the week starting at 2021-10-25, as the sixth wave wasn’t included.

### Bayesian hierarchical spatio-temporal models

We used the Bayesian hierarchical spatio-temporal framework to model the weekly observed counts of COVID-19 cases/hospitalisations, $Y_{it}$, as follows:

2 $$\begin{aligned} \begin{aligned} Y_{it} \mid \theta _{it}&\sim Poisson(E_i \theta _{it}) \\ \log {\theta _{it}}&= \alpha + b_i + \gamma _t + w_t + \delta _{it} \end{aligned} \end{aligned}$$

where $\alpha$ quantifies the global risk; $b_i$ is the spatial effect; $\gamma _t$ and $w_t$ are the temporally structured and unstructured random effects, respectively; and $\delta _{it}$ models the spatio-temporal interaction random effect. With this formulation, the maximum likelihood (ML) estimator of $\theta _{it}$ is given by $\hat{\theta _{it}} = Y_{it}/E_i$ corresponding to the SIR. Thus, the estimated $\hat{\theta _{it}}$ is a smooth estimate of the SIR and can be interpreted as the area and week specific relative risk (RR), with respect to the global territory of Catalonia for the whole period.

This modelling framework provides a flexible and robust approach that allows us to fit the risk patterns of interest by specifying different types of complex spatial, temporal and spatio-temporal structures as random effects. We considered the set of non-parametric models proposed by Knorr-Held^27^, which are widely used in space-time disease mapping and allow us to account for spatial and temporal trends as well as different scenarios of potential area-specific differences in trends. These models consider conditional autoregressive (CAR) priors for the spatial effect, random walks of first or second order for the temporal effect, and four different types of spatio-temporal interaction effects. The best model in terms of different model selection criteria was selected. We use the Deviance Information Criterion (DIC)^28^ and the Widely Applicable Information Criterion (WAIC)^29^ as model selection criteria.

In addition, this framework allows us to explore the effect of different risk factor covariates by including them in the model as fixed effects. To study the association with the socio-demographic characteristics of the ABS, we included the available covariates in the model (2) as fixed effects. The linearity assumption of the relationship between the included covariates and the outcomes was assessed by plotting the estimated spatial RR of the raw model without including them against each of the covariates and fitting a smooth curve using Local Polynomial Regression Fitting^30^. Finally, to examine the association with vaccination, we added the cumulative percentage of full vaccination in the past week to the model (2) as a fixed effect together with the previously included socio-demographic covariates. As explained before, for the vaccination analysis $E_{it}$ was calculated for each week independently, so that the estimated $\hat{\theta _{it}}$ represents the area and week specific RR with respect to the global territory of Catalonia at the same particular week. The linearity assumption of the relationship between fully vaccination and the outcome was assessed by plotting the estimated spatial RR of the raw model against the cumulative fully vaccination over the entire period.

#### Spatial effect

The spatial effect $b_i$ was first modelled using the classical Besag York Mollié (BYM)^31^:

3 $$\begin{aligned} b_i = \alpha + S_i + U_i \end{aligned}$$

where $S_i$ and $U_i$ are the spatial random effects modelling the spatial dependence structure and the spatial unstructured uncorrelated noise, respectively. Furthermore, in this model, the spatial structured term *S* is modelled using a conditional autoregressive (CAR) distribution, where the values on a given area depend on the average of the values on a small set of neighbouring areas in the following way:

$$\begin{aligned} S_i | S_{-i} \sim N({\overline{S}}_{\delta _i}, \frac{\sigma _S^2}{n_{\delta _i}}) \end{aligned}$$

where $\delta _i$ is the set of neighbours, $n_{\delta _i}$ is the number of neighbours, ${\overline{S}}_{\delta _i}$ is the average of the values in the neighbours and $\sigma ^2_S$ is the variance of the structured effect. We defined the neighbours as areas that share a common border, that is the most common and straight-forward assumption, although other more complex neighbourhood structures can be considered^32^. In contrast, the spatial unstructured effect *U* was modelled using an independent and identically distributed (IID) normal variable with zero mean and variance $\sigma _U^2$.

Because the BYM model is known to suffer from a lack of model identifiability^33^, we also modelled the spatial random effect using the BYM2 model, that is a reparametrisation of the classical BYM model^34^:

4 $$\begin{aligned} b = \sigma _b(\sqrt{\phi }S_* + \sqrt{1-\phi }U) \end{aligned}$$

where $\phi \in [0,1]$, called the mixing parameter, represents the weight of the structured effect over the unstructured one, and $\sigma _b$ is the pure standard deviation of the total spatial effect. With this new formulation, the trade-off between the unstructured and structured variation is made explicit, so that there is no longer an identifiability problem, and the new hyperparameters $\phi$ and $\sigma _b$ are interpretable and no longer confounded. We estimated both the BYM model in Eq. (3) and the BYM2 model in Eq. (4) on our data and check that the estimates obtained are similar and that the latter performs at least as well as the former with respect to DIC and WAIC criteria, as has already been seen in the literature^35^.

The spatial effect RR, $\text {RR}_{\text {Spatial}} = \exp (b_i)$, was estimated for each ABS and the posterior mean was represented in maps. This effect represents the lack/excess risk of an area compared to the general population on average over the whole period. We defined hotspots as those ABS with a posterior probability of having a spatial effect RR greater than 1 between 0.8 and 1, $0.8 \le P(\text {RR}_{\text {Spatial}} > 1) \le 1$, whereas coldspots were defined as those ABS with this probability less than or equal to 0.2, $P(\text {RR}_{\text {Spatial}} > 1) \le 0.2$. Previous to including the demographic and socio-economic factors to the model, these factors were compared between hotspots and coldspots to illustrate their potential effect in explaining differences in the ABS spatial effect RR. For the vaccination analysis, vaccination percentages over the whole period were also compared between hotspots and coldspots.

#### Temporal effect

The temporal structured effect $\gamma _t$ was modelled assuming either a first-order random walk (RW1) imposing a dependency on the previous week:

$$\begin{aligned} \gamma _t | \gamma _{t-1} \sim N(\gamma _{t-1}, \sigma _{\gamma }^2) \end{aligned}$$

or a second-order random walk (RW2) imposing a dependency in the two preceding weeks:

$$\begin{aligned} \gamma _t | \gamma _{t-1}, \gamma _{t-2} \sim N(2\gamma _{t-1} + \gamma _{t-2}, \sigma _{\gamma }^2) \end{aligned}$$

The temporal unstructured effect $w_t$ was modelled as an IID normal random variable with mean 0 and variance $\sigma _w^2$. In practice, this effect is seen to be unnecessary in most cases^36^, so we replicated the model without it and assessed that the performance was similar.

The temporal effect RR, $\text {RR}_{\text {Temporal}} = \exp (\gamma _t)$, was estimated for each week and the posterior mean was plotted. This effect represents the lack/excess risk for the general population of one week compared to the average for the whole period.

#### Spatio-temporal interaction effect

The interaction effect $\delta _{it}$ followed a normal distribution with a precision matrix given by $\tau _{\delta }R_{\delta }$, where $\tau _{\delta }$ is the precision of the effect (inverse of the variance) and $R_{\delta }$ is the structure matrix identifying the type of temporal and spatial dependence between the elements of $\delta _{it}$. This structure matrix can be factorised as the Kronecker product of the structure matrix of the corresponding random effects interacting. As proposed by Knorr-Held^27^, we estimated different types of interaction effects by combining different pairs of spatial and temporal effects in Eq. (2):

- Type I: the spatial unstructured effect *U* interacts with the temporal unstructured effect *w*. It assumes that there is no structure in the spatio-temporal interaction ($R_{\delta } = I$).
- Type II: the spatial unstructured effect *U* interacts with the temporal structured effect $\gamma$. It assumes that each area has a temporal random walk that is independent from the others ($R_{\delta } = I \otimes R_{\gamma }$).
- Type III: the temporal unstructured effect *w* interacts with the spatial structured effect *S*. It assumes that in each week there is a spatial CAR distribution independent from the others ($R_{\delta } = I \otimes R_S$).
- Type IV: the spatial structured effect *S* interacts with the temporal structured effect $\gamma$. It assumes that the temporal trend in an area is similar to the average trend in the neighbouring areas ($R_{\delta } = R_S \otimes R_{\gamma }$).

The spatio-temporal effect RR, $\text {RR}_{\text {Spatio-temporal}} = \exp (\delta _{it})$, was estimated for each ABS and week and the posterior mean was plotted. This effect represents the lack/excess risk of one week and ABS compared to the average for the general population over the whole period that remains unexplained after adjusting for the spatial and temporal effect alone.

We explored all the different types of spatio-temporal models presented in this chapter to see which ones better fit the data in terms of DIC and WAIC. The best model was chosen to estimate the spatial, temporal and spatio-temporal patterns. The analysis of the effect of spatial risk factors and vaccination was then performed on the basis of the best selected model.

The models were fitted using INLA^37^, which is an alternative to classical Markov chain Monte Carlo (MCMC), for approximating Bayesian inference performed on latent Gaussian models (a subclass of structured additive regression models), including a wide range of models from generalised linear mixed models to spatial and spatio-temporal models such as those used in this study. These models often involve dealing with sparse precision matrices, and INLA takes advantage of this to speed up computation. We had to consider some additional issues in order to fit these models:

#### Linear constraints

In the context of spatio-temporal models, identifiability problems can arise because the model intercept can be absorbed by both the spatial and temporal effects, and the interaction terms can be confounded with the main effects^27^. To ensure model identifiability, we imposed sum-to-zero constraints on the main spatial and temporal random effects^38^. For the spatial effect, the sum-to-zero constraint was $\sum _{i=1}^{n} b_i = 0$, while for the temporal effect it was $\sum _{t=1}^{T} \gamma _t = 0$ assuming a RW1 distribution. Otherwise, for the spatio-temporal interaction effect, the identifiability constraints to be imposed depended on the type of effect presented earlier:

- Type I: $$\sum \limits _{i=1}^{n} \sum \limits _{t=1}^{T} \delta _{it} = 0$$
- Type II: $\sum \limits _{t=1}^{T} \delta _{it} = 0$, for $i= 1,...,n$
- Type III: $\sum \limits _{i=1}^{n} \delta _{it} = 0$, for $t= 1,...,T$
- Type IV: $\sum \limits _{t=1}^{T} \delta _{it} = 0$, for $i= 1,...,n$; $\sum \limits _{i=1}^{n} \delta _{it} = 0$, for $t= 1,...,T$

#### Prior distributions

When performing Bayesian inference, the choice of prior distribution plays a crucial role because it encapsulates the information available for the parameters of interest in the model and can affect the final results.

For the BYM spatial model, the hyperparameters are the standard deviation of the structured and unstructured random effects $\sigma _S$ and $\sigma _U$. As we don’t had any prior information on the hyperparameters of the models, we used non-informative priors. For hierarchical standard deviation hyperparameters, it is recommended to use uniform priors rather than the gamma family priors that are commonly used^39^. The main inconvenience of the latter prior is that when the estimated hyperparameter values are close to zero, the inference becomes very sensitive to the choice of parameters of the gamma prior distribution, and it hardly looks non-informative. Therefore, an improper uniform prior distribution on the positive real line $U(0, \infty )$ was chosen for $\sigma _S$ and $\sigma _U$.

For the BYM2 spatial model, recall that the hyperparameters are the marginal standard deviation $\sigma _b$ and the mixing parameter $\phi$. Now that these parameters are interpretable, it is easier to assign meaningful Penalised Complexity (PC) priors. This family of priors penalises model complexity in terms of the deviation from the flexible model to the base model, which has a constant RR over all areas. We defined these PC priors using probability statements on the model hyperparameters at the appropriate scale. For the marginal standard deviation parameter, $\sigma _b$, the PC prior was defined by the parameters *U* and *a* such that $P(\sigma _b > U) = a$. Considering a value of 0.5 as a reasonable upper bound for the marginal standard deviation, using the rule of thumb^34^, we set $U = 0.5/0.31$ and $a = 0.01$. The values of the PC prior for the mixing parameter $\phi$ were inferred in a similar way, now using the probability statement $P(\phi < U) = a$. We set $U = 0.5$ and $a = 2/3$, which is a conservative choice that assumes that the unstructured random effect accounts for more of the variability than the spatially structured effect^24^.

In the spatio-temporal models, the uniform prior $U(0, \infty )$ was chosen for the standard deviation hyperparameters of the temporal random effect ($\sigma _{\gamma }$, $\sigma _w$) and the spatio-temporal interaction effect ($\sigma _{\delta }$), using the same reasoning as above for the BYM model.

### Software

All code was developed using the free statistical software R in the version 4.3.0^40^. The main R package used was R-INLA^41^ to fit the models with INLA. These references were followed as guidelines on how to perform spatio-temporal analysis using R and R-INLA^24,42,43^. Other than R-INLA, the R packages dplyr^44^ and purrr^45^ were used for data management and ggplot2^46^ for visualization. All implemented code used in this study is publicly available online at:

https://github.com/pasahe/Bayesian-spatio-temporal-analysis-of-COVID-19-in-Catalonia..

## Results

### Description of the COVID-19 pandemic in Catalonia

**Figure 2:**
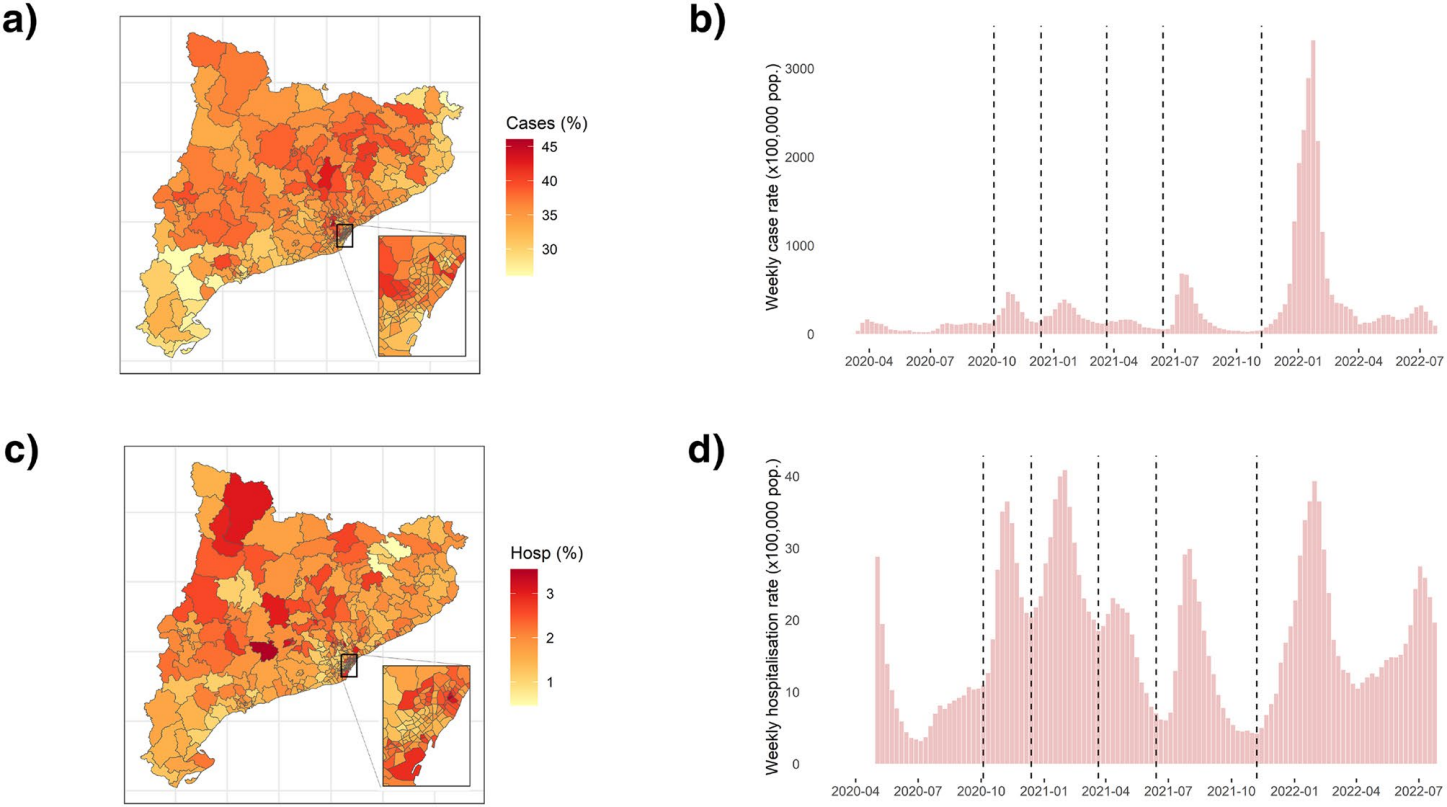
COVID-19 cases and hospitalisation weekly rates and cumulative distribution by basic health areas (ABS). (**a**) Map of the total cumulative incidence percentage of COVID-19 cases over the whole study period for each ABS. (**b**) Raw rate (×100,000 population) of COVID-19 cases in Catalonia per week. (**c**) Map of the total cumulative incidence percentage of COVID-19 hospitalisations over the whole study period for each ABS. (**d**) Raw rate (×100,000 population) of COVID-19 hospitalisations in Catalonia per week. Figures generated in R version 4.3.0.

During the study period, a cumulative total of 2,685,568 COVID-19 cases and 144,550 hospitalisations were reported in Catalonia, representing a 35% and a 1.89% of the total population. In Fig. 2b and d the evolution of weekly cases and hospitalisation rates (×100,000 population) in Catalonia over the whole study period is represented. The vertical dotted lines represent the start of each of the six waves, which were very different in shape, especially for cases. Considering all the pandemic period, the proportion of the total population infected ranged from a minimum of 26% in some areas to a maximum of 46% in others, while the proportion of the total population hospitalised ranged from 0.5% to 3.5% (Fig. 2a and c).

### Spatio-temporal models

Different base spatio-temporal models were estimated and their DIC and WAIC values are presented in Table 1. For the spatial effect, the BYM2 model fitted the data better than the BYM (lower DIC and WAIC), for cases and hospitalisations. For the temporal effect, the model without the unstructured temporal effect fitted the data better. Finally, for the structured temporal effect, RW2 fitted the data better although the differences were meaningless. For the spatio-temporal effect, the model with type II interaction had the lowest DIC and WAIC values for the cases outcome and similar values to type IV for the hospitalisations. Therefore, the base model chosen was the spatial and temporal structured effect modelled with BYM2 and RW1 respectively, no unstructured temporal effect and the spatio-temporal interaction effect modelled with type II.

**Table 1:** Base spatio-temporal model selection.

(a) BYM versus BYM2 model for the spatial effect				
	Cases		Hospitalisations	
	DIC	WAIC	DIC	WAIC
BYM	316,747.2	313,005.8	164,898.1	164,964.1
BYM2	316,543.7	312,578.1	164,837.2	164,949.2

(b) Including the temporal unstructured effect versus not including it				
	Cases		Hospitalisations	
	DIC	WAIC	DIC	WAIC
Temp. unstruc.	316,655.0	312,879.0	164,914.4	164,963.6
No temp. unstruc.	316,543.7	312,578.1	164,837.2	164,949.2

(c) RW1 versus RW2 for the temporal structured effect				
	Cases		Hospitalisations	
	DIC	WAIC	DIC	WAIC
RW1	316,543.7	312,578.1	164,837.2	164,949.2
RW2	316,522.2	312,539.6	164,813.9	164,857.5

(d) Different types of spatio-temporal interaction effect				
Interaction	Cases		Hospitalisation	
	DIC	WAIC	DIC	WAIC
I	316,543.7	312,578.1	164,837.2	164,949.2
II	309,233.2	310,396.0	149,779.0	147,911.7
III	313,425.1	311,657.3	161,219.4	161,330.7
IV	310,456.4	314,764.5	149,623.4	148,345.7

Estimated Deviance Information Criterion (DIC) and Widely Applicable Information Criterion (WAIC) values by the different spatio-temporal models with different specifications for each of the random effects.

**Table 2:** Spatio-temporal model summary.

(a) Hyperparameters of the model.					
	Intercept	SD (idarea)	Phi (idarea)	SD (idtime)	SD (idareatime)
Cases	0.44 (0.44, 0.44)	0.15 (0.14, 0.16)	0.84 (0.79, 0.89)	0.45 (0.38, 0.54)	0.28 (0.27, 0.28)
Hospitalisations	0.69 (0.68, 0.7)	0.31 (0.28, 0.34)	0.55 (0.44, 0.63)	0.19 (0.17, 0.22)	0.2 (0.2, 0.21)

(b) Proportion percentages of the total variability explained by each random effect.					
	Variance spatial (%)	Variance temporal (%)	Variance spatio-temporal (%)		
Cases	7.21	67.51	25.28		
Hospitalisation	54.38	21.79	23.82		

Estimated hyperparameters and proportions of total variability explained by the spatial, temporal and spatio-temporal random effects for the spatio-temporal model.

The estimated values for the hyperparameters of the model with type II spatio-temporal interaction model are presented in Table 2, together with the percentage of variability explained by each spatial, temporal and spatio-temporal component. The estimated $\phi$ value was greater than 0.5, so the proportion of the spatial variance explained by the structured effect was greater than that explained by the unstructured effect for both outcomes, being greatest for cases. The temporal effect played the greatest role in explaining the total variability for cases, whereas the spatial effect was the largest for hospitalisations.

**Figure 3:**
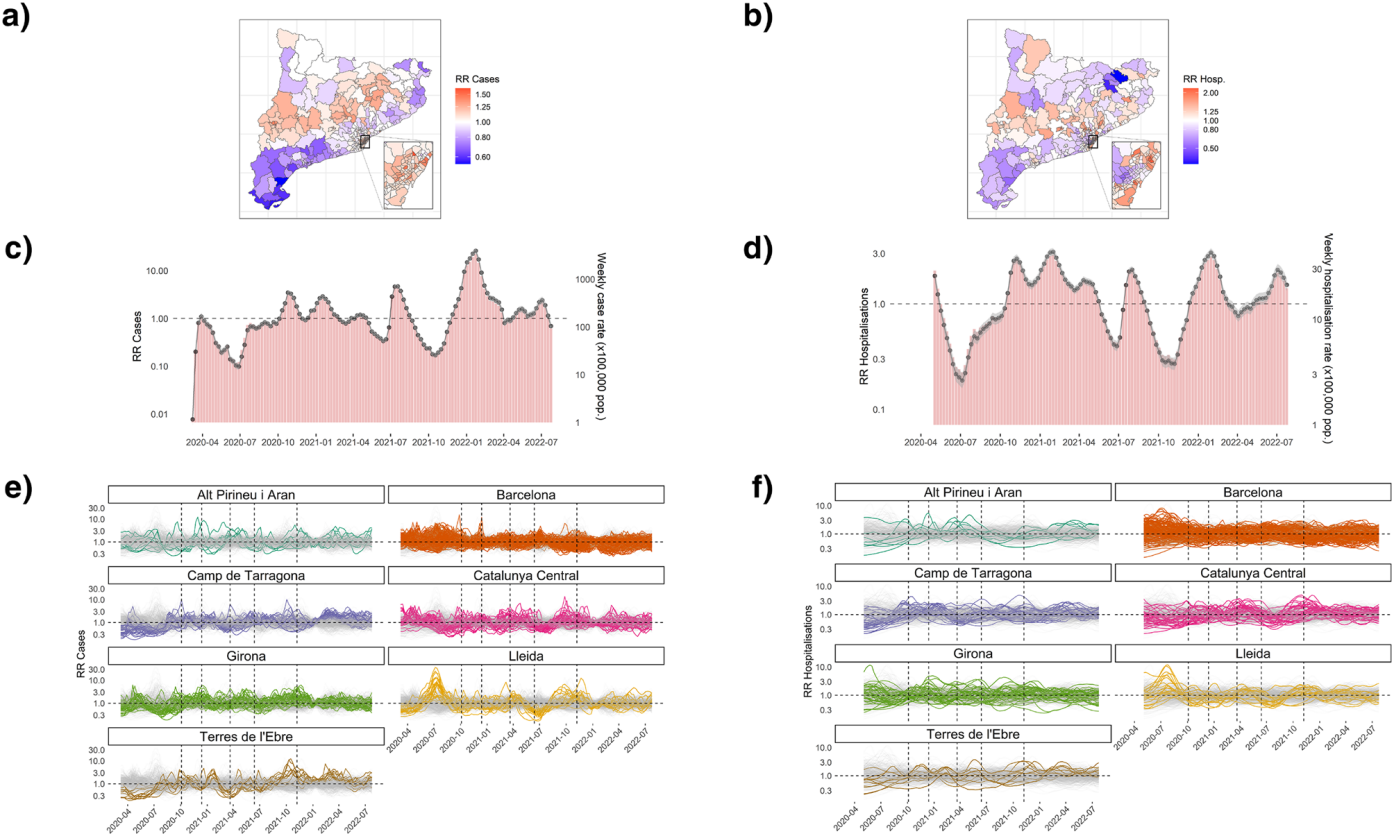
Spatial, temporal and spatio-temporal relative risks (RR) estimated by the spatio-temporal model. (**a**) Map of the posterior mean estimates of the marginal spatial RR of COVID-19 cases for each basic health area (ABS). (**b**) Map of the posterior mean estimates of the marginal spatial RR of COVID-19 hospitalisations for each ABS. (**c**) Line plot of the posterior mean estimates of the marginal temporal RR of COVID-19 cases, together with the bar plot of the weekly raw rate (×100,000 population) of COVID-19 cases in Catalonia, per week. Values are presented on a logarithmic scale. (**d**) Line plot of the posterior mean estimates of the marginal temporal RR of COVID-19 hospitalisations, together with the bar plot of the weekly raw rate (×100,000 population) of COVID-19 hospitalisations in Catalonia, per week. Values are presented on a logarithmic scale. (**e**) Posterior mean estimates of the marginal spatio-temporal RR of COVID-19 cases. For each region, the ABS within it are highlighted in colour. Values are presented on a logarithmic scale. (**f**) Posterior mean estimates of the marginal spatio-temporal RR of COVID-19 hospitalisations. For each region, the ABS within it are highlighted in colour. Values are presented on a logarithmic scale. Figures generated in R version 4.3.0.

The estimated spatial patterns of reported COVID-19 cases and hospitalisations are shown in Fig. 3a and b, respectively. The RRs obtained for each area ($\text {RR}_{\text {Spatial}}$) were clustered in different regions. For cases, there was a strong clustering of hotspots in Barcelona, in Lleida, in some areas of Catalunya Central and in areas close to Vic. Conversely, there was a strong clustering of coldspots in all the areas in the south of the territory (Terres de l’Ebre and Tarragona) and in the areas along the northeast coast. Hospitalisation risks were also clustered in these former regions, but there was more variability in the areas within them, with the exception of Terres de l’Ebre, which remained a uniform cluster of coldspots. In Barcelona, for example, there were both coldspots and hotspots, so areas that were close together had more variability than for cases.

The estimated temporal patterns of the reported COVID-19 cases and hospitalisations are shown in Fig. 3c and d. The RRs obtained for each week ($\text {RR}_{\text {Temporal}}$) fitted very well the evolution of the incidence rates for the whole of Catalonia .

The estimated spatio-temporal patterns of the reported COVID-19 cases and hospitalisations are shown in Fig. 3e and f. For each region, the RRs obtained for each area within the region and week ($\text {RR}_{\text {Spatio-Temporal}}$) are highlighted in colour. For both cases and hospitalisations, the highest spatio-temporal interaction effect between the first and second wave were in the areas of Lleida. For hospitalisations, there was also an area in Girona that had a very high interaction effect at the beginning of the first wave, and in some areas in Barcelona between the first and second waves. Also for Terres de l’Ebre and Tarragona, the pattern of spatio-temporal effects in their areas was very similar for both cases and hospitalisations, characterised by low values in the first wave and high values in the last reported wave. Moreover, in all the regions there were many areas that deviate from the overall temporal trend of the whole territory at many different points in time, either having higher spatio-temporal effects (excess of risk) or lower spatio-temporal effects (lack of risk).

### Association with spatial covariates

For cases, there were virtually no differences in socio-economic index values between hotspots and coldspots, given by the spatial effect of the last spatio-temporal model (Supplementary Fig. S2). Conversely, for hospitalisations, hotspots had the highest values and coldspots the lowest.

The model could not be adjusted for all the different socio-economic components at the same time because some of them were highly correlated ($\rho > 0.7$, Supplementary Fig. S3). The linearity of the relationships of each of the included variables with cases and hospitalisations was also assessed (Supplementary Figs. S4 and S5). All the relationships were linear, except for the socio-economic index, which had a quadratic association with cases. Therefore, the square of the socio-economic index was included in the cases model to account for this quadratic effect.

**Table 3:** Spatio-temporal model coefficients, including demographic and socio-economic variables.

(a) Cases			
	Raw	Urban + socio-economic index	Urban + socio-economic components
Fixed effects			
(Intercept)	0.44 (0.44, 0.44)	0.42 (0.41, 0.43)	0.43 (0.42, 0.44)
Urban vs rural		1.07 (1.04, 1.11)	1.05 (1.02, 1.09)
Socio-economic Index		0.99 (0.97, 1)	
Socio-economic Index⌃2		1.02 (1.01, 1.02)	
Pharmaceutical co-payment			1.01 (0.99, 1.03)
Income < 18,000€			0.97 (0.95, 0.99)
Inadequate education			1 (0.98, 1.02)
Premature mortality			1 (0.99, 1.02)
Avoidable hospitalisations			1 (0.99, 1.02)
Random effects			
Standard deviation (idarea)	0.15 (0.16, 0.14)	0.11 (0.12, 0.11)	0.12 (0.13, 0.11)
Phi for idarea	0.84 (0.79, 0.89)	0.18 (0.14, 0.25)	0.27 (0.22, 0.35)
Standard deviation (idtime)	0.45 (0.54, 0.38)	0.44 (0.49, 0.4)	0.46 (0.55, 0.41)
Standard deviation (idareatime)	0.28 (0.28, 0.27)	0.28 (0.28, 0.27)	0.28 (0.28, 0.28)

(b) Hospitalisations			
	Raw	Urban + socio-economic index	Urban + socio-economic components
Fixed effects			
(Intercept)	0.69 (0.68, 0.7)	0.64 (0.61, 0.66)	0.65 (0.63, 0.68)
Urban vs rural		1.17 (1.1, 1.25)	1.12 (1.05, 1.21)
Socio-economic Index		1.19 (1.17, 1.22)	
Pharmaceutical co-payment			1.09 (1.05, 1.13)
Income < 18,000€			1.11 (1.07, 1.16)
Inadequate education			0.99 (0.95, 1.03)
Premature mortality			1.01 (0.98, 1.03)
Avoidable hospitalisations			1.04 (1, 1.07)
Random effects			
Standard deviation (idarea)	0.34 (0.36, 0.31)	0.22 (0.25, 0.2)	0.23 (0.25, 0.21)
Phi for idarea	0.75 (0.61, 0.88)	0.13 (0.1, 0.17)	0.58 (0.49, 0.66)
Standard deviation (idtime)	0.2 (0.22, 0.17)	0.21 (0.24, 0.18)	0.2 (0.23, 0.17)
Standard deviation (idareatime)	0.21 (0.21, 0.2)	0.2 (0.21, 0.19)	0.2 (0.21, 0.2)

Relative risks (RR) of the fixed effects and random effects hyperparameters of the different fitted spatio-temporal models for cases and hospitalisations: raw model, model including the urbanity and the socio-economic index, model including the urbanity and the socio-economic components.

Table 3 shows the estimated fixed effects of each of the included covariates together with the estimated hyperparameters of the random effects. For cases, the socio-economic index had a small quadratic effect, representing a slight increase in risk in areas with the lowest or highest values of the index. Moreover, only urban-rural and income < 18,000 euros had a small effect on the risk of cases. For urban areas there was a 5% (C.I. 2–9%) increase in risk compared to rural areas, while there was a 3% (C.I. 1–5%) decrease in risk for a one standard deviation increase in the income < 18,000 euros socio-economic component. For the risk of hospitalisation, there was a 19% (C.I. 17–22%) increase in risk for a one standard deviation increase in the socio-economic index. For all the socio-economic components, there was also a substantial effect for pharmaceutical co-payments (9%, C.I. 5–13%), income < 18,000 euros (11%, C.I. 7–16%) and avoidable hospitalisations (4%, C.I. 0–7%). Urban areas also had a substantial effect on hospitalisations after adjustment for the socio-economic index (17%, C.I. 10–25%) and also after adjustment for the socio-economic components (12%, C.I. 5–21%).

To assess whether the inference changes when the covariates are included in the final spatio-temporal model, we re-estimated the models using different structures for the spatial (BYM versus BYM2) and temporal random effects (including the temporal structured effect or modelling the structured effect as RW1 versus RW2). The comparison of the estimated DIC & WAIC values is presented in Supplementary Table S2. Differences in model performance were meaningless.

### Association with vaccination

**Figure 4:**
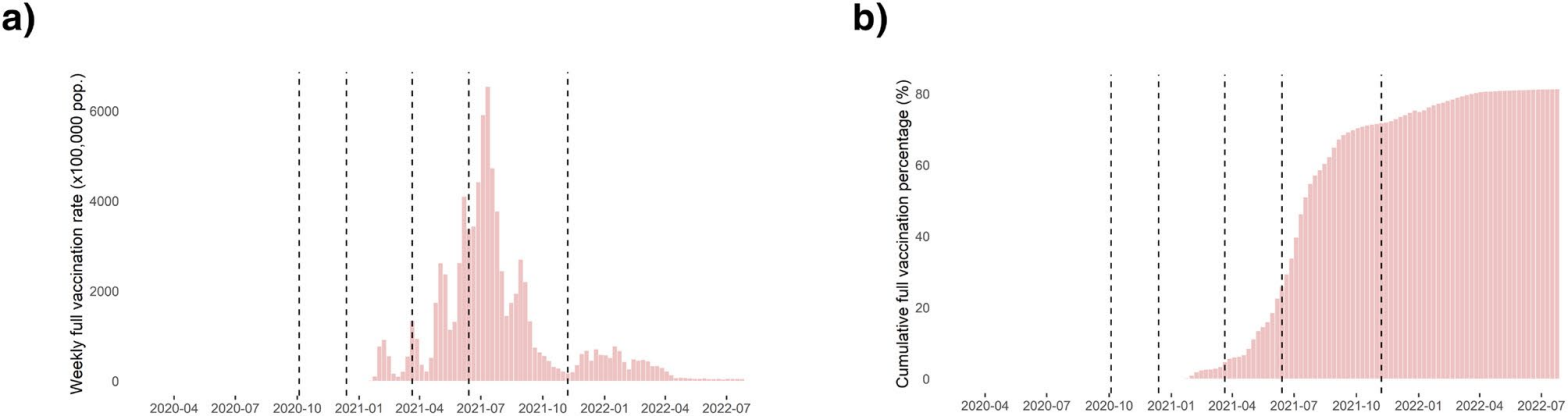
Full vaccination weekly and cumulative rates evolution per week. (**a**) Weekly raw rate (×100,000 population) of full vaccination per week in Catalonia. (**b**) Cumulative raw percentage of full vaccination per week in Catalonia. Figures generated in R version 4.3.0.

Figure 4 shows the evolution of the weekly vaccination rate (×100,000 population) and the cumulative percentage of fully vaccinated people in Catalonia. At the end of the fifth wave, more than 70% of the population was fully vaccinated.

For the third and fourth waves, hotspots had slightly higher cumulative percentages of full vaccination, given by the estimated spatial effect of the raw models (Supplementary Fig. S6). For the fifth wave, hotspots had lower values and coldspots had higher values (Supplementary Fig. S7). The linearity of the relationship between cumulative full vaccination and outcomes was also assessed (Supplementary Figs. S8 and S9).

**Table 4:** Spatio-temporal model coefficients, including demographic, socio-economic and vaccination variables.

(a) Waves 3-4		
	Hospitalisations	
Fixed effects		
(Intercept)	0.72 (0.66, 0.78)	
Urban vs rural	1.28 (1.1, 1.48)	
Socio-economic Index	1.15 (1.08, 1.21)	
Full vaccination (1-week lag)	0.94 (0.88, 1)	
Random effects		
Standard deviation (idarea)	0.48 (0.54, 0.43)	
Phi for idarea	0.62 (0.42, 0.82)	
Standard deviation (idtime)	0 (0.03, 0)	
Standard deviation (idareatime)	0.26 (0.28, 0.24)	

(b) Wave 5		
	Cases	Hospitalisations
Fixed effects		
(Intercept)	0.87 (0.84, 0.9)	0.75 (0.69, 0.81)
Urban vs rural	1.04 (0.98, 1.1)	1.19 (1.05, 1.36)
Socio-economic Index	1 (0.98, 1.03)	1.2 (1.13, 1.28)
Full vaccination (1-week lag)	0.88 (0.82, 0.95)	0.83 (0.68, 0.98)
Random effects		
Standard deviation (idarea)	0.2 (0.22, 0.18)	0.41 (0.46, 0.37)
Phi for idarea	0.47 (0.31, 0.65)	0.37 (0.14, 0.62)
Standard deviation (idtime)	0.05 (0.25, 0.03)	0.04 (0.09, 0.02)
Standard deviation (idareatime)	0.29 (0.3, 0.28)	0.23 (0.24, 0.21)

Relative risks (RR) of the fixed effects and random effects of the spatio-temporal model for hospitalisations in waves 3-4 and for cases and hospitalisations in wave 5, including the urbanity, the socio-economic index and 1-week lagged full vaccination.

Table 4 shows the estimated coefficients of the fixed effects of each explanatory variable in the adjusted model, together with the estimated hyperparameters of the random effect on hospitalisations. For the third wave, a one standard deviation increase in the cumulative percentage of full vaccination in the previous week was associated with a 6% (C.I. 0–12%) reduction in the risk of hospitalisation, after adjusting for urban-rural and the socio-economic index. For the fourth and fifth waves, a one standard deviation increase in the cumulative percentage of full vaccination in the previous week was associated with a 12% (C.I. 5–18%) substantial reduction in the risk of cases and 17% (C.I. 2–32%) reduction in the risk of hospitalisations, after adjusting for the urban-rural and socio-economic index.

## Discussion

This work provides a comprehensive study to understand the COVID-19 pandemic across the territory of Catalonia at a small area level, describing the spatial, temporal and spatio-temporal patterns of the disease. Urban areas were found to have a higher risk of COVID-19 cases and hospitalisations compared to rural areas, while socio-economic deprivation of the area was a risk factor for hospitalisations. Full vaccination coverage was also shown to have a protective effect on the risk of COVID-19 cases and hospitalisations in the different ABS in specific waves of the pandemic.

Over time, we identified six different waves over all the study period in Catalonia. The temporal pattern of the reported COVID-19 cases had a very high variability between the different waves (Fig. 2a) mainly due to the difference in virus variants and in diagnostic capacity and effort, since in the beginning there was a low testing capacity and at the end of the sixth wave only vulnerable cases were reported due to a change in diagnostic policy. Changes in diagnostic testing practices is an inherent limitation of the study of COVID-19 transmission that has been studied in literature^47,48^, so interpretation of results obtained for cases must be made with caution. The temporal pattern of the reported COVID-19 hospitalisations, on the other hand, is more comparable between waves, although hospitalisations could also be underestimated in periods of hospital congestion due to high demand for beds, especially in the first wave. In the estimated spatio-temporal models, the variability explained by the temporal effect was higher for cases than for hospitalisations due to this greater amount of variability throughout time for cases (Table 2b). The general temporal trend of the pandemic was well captured by using a structured effect assuming a RW1 (Fig. 3c and d). RW1 it’s a common choice for modelling the temporal effect in other studies that model the COVID-19 temporal trend^7,8^.

If we take the whole pandemic period at once, the spatial patterns of reported COVID-19 cases and hospitalisations were heterogeneously distributed by ABS (Fig. 2a and c). In the estimated spatio-temporal model, the range of spatial RR estimates was wider for hospitalisations than for cases (Fig. 3a and b), as the proportion of variability explained by the spatial effect was higher (Table 2b), and closer areas generally had more similar RR values for cases than for hospitalisations, because the role of the spatially structured effect, given by the value of $\phi$ was higher for cases. For example, in Barcelona there were low-risk and high-risk areas for hospitalisations, whereas for cases there was a fairly uniform cluster of high-risk areas. Estimated values of the mixing parameter were very similar to those obtained in another study that also uses a spatio-temporal model with BYM2 to model COVID-19 cases and deaths in the United States^8^. The weight of the spatial structured effect over the total spatial effect, given by the mixing parameter $\phi$, was found to be of 82.7% for cases, very similar to the 84% obtained in this study, while this value was lower for the most severe outcome, mortality, at 60.9%, which is similar to the 55% obtained for hospitalisations in this study (Table 2a). The higher $\phi$ obtained for COVID-19 cases could be due to the fact that infectious diseases are generally more likely to spread in nearby areas, so COVID-19 infections were more likely to occur in adjacent areas. Conversely, hospitalisations, which occur when individuals with more severe symptoms require medical attention and admission to hospital, depend on several factors beyond the spread of the virus, so adjacent areas with different conditions may have presented different risks. In this work, we assumed a spatial structured dependence based on adjacent geographical boundaries, but we could specify more complex weighted matrices modulating the strength of the dependence of nearby pairs of areas to reflect other similarity features, not just sharing the same border^49^.

The spatio-temporal patterns of reported COVID-19 cases and hospitalisations were heterogeneously distributed by ABS and time. Throughout time, some ABS had periods with lower/higher incidence values that were different from other ABS (Fig. 3e and f). The most prominent example was the outbreak in the region of Lleida between the first and second waves, a period characterised by the arrival of large numbers of seasonal farmers, which, according to the literature, could be related to COVID-19 outbreaks^50^. Among the different types of spatio-temporal interaction effects, the one that best fitted the data was the type II (Table 1d), which assumes a RW1 for each area independent of the others. There are other spatio-temporal studies of COVID-19 that also found type II as the model that best fits their data^7–9,51^.

Of all the explored factors, the socio-economic index had the highest risk effect on hospitalisations (Table 3b). Among the different socio-economic components that make up the index, those with a substantial risk effect were income < 18,000 euros, pharmaceutical co-payment and avoidable hospitalisations. Urban areas also had a substantial risk effect, adjusted for these components. These findings were consistent with other studies showing the negative impact of low socio-economic status^8,9,52,53^ and higher population density^54,55^ on severe COVID-19 outcomes such as hospitalisation or mortality.

However, for COVID-19 cases most of these spatial covariates did not have a substantial effect in the model (Table 3a). The socio-economic index had a small quadratic effect, with the lowest and highest socio-economically deprived ABS having slightly higher risks than the rest. Of the other covariates, only urban areas had a small risk effect adjusted by the socio-economic components, while income < 18,000 euros had a small protective effect. Urbanicity favours the spread of the virus, as urban areas have a higher population density that is a well studied transmission factor of the spread of the virus^10,56–58^. For the socio-economic variables, some of the reviewed studies show that a lower socio-economic status is associated with a higher risk of COVID-19 infection^8,10,57^, although it has also been seen to be associated with lower testing^59,60^, which could directly affect the number of reported COVID-19 cases. Therefore, the small effects of the socio-economic variables found for the reported COVID-19 cases in this study could be due to the association with lower testing offsetting the association with higher COVID-19 infection, thus compensating for each other. For example, in another study analysing differences in confirmed COVID-19 cases, hospitalisations and deaths in another region of Spain, Andalusia, a protective effect of the income for infection was only observed in 3/12 cities, compared with 10/12 for hospitalisations and 8/12 for deaths^52^.

Full vaccination in the previous week had a small protective effect on hospitalisations in the third and fourth waves for the population aged 70 and over (Table 4a). In the fifth wave, for the whole population, the protective effect was greater for cases, and even greater for hospitalisations (Table 4b). These results support the extensive research on the protective effect of COVID-19 vaccination, especially in the development of severe COVID-19 disease^11–14^.

One of the limitations of this study was the lack of more available covariates that could play a role in explaining COVID-19 and confound some of the observed effects. The dynamics of COVID-19 are very complex and the literature shows a very wide range of different factors influencing them. For example, demographic factors such as ethnicity and immigration status, environmental factors such as air pollution, temperature and humidity, healthcare resources such as the number of medical practitioners and hospital beds or specific containment strategies such as lockdown have been identified as drivers of the pandemic^5,15,61^. Many of these indicators are not easily available in Catalonia, especially at the ABS level. For our study, it was not possible to obtain any of them and their possible relationship with the incidence of cases and hospitalisations could not be explored. In any case, they would be of great interest for future work. It would also be interesting to study the impact of these factors on COVID-19 deaths, as has been done in the literature, and to study the spatio-temporal distribution of this outcome in Catalonia, as we did for cases and hospitalisations, but COVID-19 mortality data from ABS were not publicly available.

The vaccination study also had some limitations. First, we considered the entire history of vaccination by taking cumulative counts. This is a simplistic approach that assumes that the effect of vaccination does not diminish over time and also reduces the spatial variability of this variable. In addition, we examined the effect of vaccination in explaining the spatial and spatio-temporal effect for cases and hospitalisations, but not the temporal effect, because we modelled the SIR calculated using expected counts in each week. Thus, differences in observed outcomes in the whole territory between different time periods, which could be due to an increase in vaccination, were not explored. We only assessed the effect of vaccination on the risk differences by ABS in each week, given by the spatio-temporal interaction effect, and on the risk differences by ABS over the whole period, given by the spatial effect. Finally, the sixth wave was excluded from the analysis, but it would be interesting to study the effect that vaccination might have had in this wave taking into account the booster shots that were given mainly to the most vulnerable population during this period.

## Conclusions

In this study we explored the COVID-19 pandemic across the territory of Catalonia at a small area level, describing the spatial, temporal and spatio-temporal trends of the disease. We also provided insight into some of the factors associated with COVID-19, showing that urban areas have a higher risk of COVID-19 cases and hospitalisations compared to rural areas, while socio-economic deprivation of the area was a risk factor for hospitalisations. Bayesian hierarchical modelling was found to be very useful for this task, providing a flexible and robust framework. This study contributes to the literature exploring the spatio-temporal pattern and factors associated with COVID-19 in small area-level studies in other regions of the world.

### Supplementary Information

Supplementary Information.

## Data Availability

The datasets used during the current study are available in the official open data catalogue of the Government of Catalonia, https://analisi.transparenciacatalunya.cat/en/. The datasets generated during the study are available in a github repository, https://github.com/pasahe/Bayesian-spatio-temporal-analysis-of-COVID-19-in-Catalonia.
