# Supplementary material for: Bayesian spatio-temporal analysis of the COVID-19 pandemic in Catalonia
Source: Sci Rep. 2024 Feb 20;14:4220. doi: 10.1038/s41598-024-53527-w (PMC10879174; doi:10.1038/s41598-024-53527-w)

Supplementary Information

Supplementary Table S1. List of the included demographic and socio-economic ABS characteristics in the study.

Covariates	Definition
Urban-rural indicator	Indicator showing whether an ABS is urban or rural. It is considered urban if it is located in a city with more than 3 ABS, or with 2 ABS if one of them has a population density of more than 10,000 inhabitants per square kilometre.
Socio-economic index	Score representing the ABS socio-economic deprivation. It is a single index composed by different socio-economic indicators aggregated by weights[1].
**Socio-economic index components (standardised by age)**	
Population exempted from pharmaceutical co-payment	Percentage of the ABS population exempted from pharmaceutical co-payment. Population exempted are those individuals in an economic vulnerable situation[2].
Population with income < 18,000 euros	Percentage of the ABS population with an annual income of less than 18,000 euro per person.
Population with income > 100,000 euros	Percentage of the ABS population with an annual income of more than 100,000 euro per person.
Population with manual employment	Percentage of the ABS population working in manual occupations.
Population with inadequate level of education	Percentage of the ABS population with an inadequate level of education. Level of education is considered inadequate if the individual does not know how to read or write, or has only a primary education.
Premature mortality	Premature mortality ABS rate. Premature mortality is defined as a death that occurs before the age of 75 years.
Avoidable hospitalisations	Potential avoidable hospitalisations. Hospitalisation admissions are considered avoidable if the diagnose is an ambulatory care sensitive condition[3].

[1] https://observatorisalut.gencat.cat/en/observatori-desigualtats-salut/dades_obertes/index.html#new-socioeconomic-indicator-for-abs-financing

[2] Groups exempted from contribution can be consulted in: https://catsalut.gencat.cat/ca/serveis-sanitaris/atencio-farmaceutica/financament-public-medicaments/model-copagament/index.html#googtrans(calen)

[3] Acute or chronic health issues that lead to potentially preventable hospitalizations when not treated in the outpatient primary care setting.

Supplementary Figure S1. Urban-rural indicator and socio-economic index map. Map of the urban-rural indicator and the values of the socio-economic index for each basic health area (ABS). Figures generated in R version 4.3.0.

(a) Urban-rural indicator **(b)** Socio-economic index

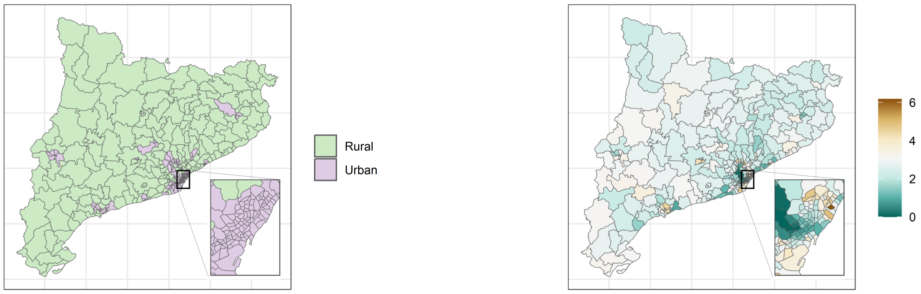

Supplementary Table S2. Spatio-temporal model with covariates and different models for the spatial and temporal random effects. Estimated Deviance Information Criterion (DIC) and Widely Applicable Information Criterion (WAIC) values for the different spatio-temporal models adjusted by the urban-rural index and the socio-economic components, with different specifications for each of the random effects.

(a) BYM versus BYM2 model for the spatial effect.

	Cases		Hospitalisations	
	DIC	WAIC	DIC	WAIC
BYM	309303.7	310293.5	149741.5	147910.6
BYM2	309304.1	310294.7	149746.3	147904.0

(b) Including the temporal unstructured effect versus not including it.

	Cases		Hospitalisations	
	DIC	WAIC	DIC	WAIC
Temporal unstructured	309303.3	310292.1	149741.4	147910.6
No temporal unstructured	309303.2	310291.9	149745.8	147901.5

(c) RW1 versus RW2 for the temporal structured effect.

	Cases		Hospitalisations	
	DIC	WAIC	DIC	WAIC
RW1	309303.6	310292.9	149742.1	147906.0
RW2	309379.3	310579.6	149740.3	147900.6

Supplementary Figure S2. Socio-economic differences between hotspots/coldspots. Boxplot of socio-economic index values between hotspots and coldspots given by the spatial effect of the raw spatio-temporal model for cases and hospitalisations. Areas that cannot be defined as either hotspots or coldspots are shown in white. Figures generated in R version 4.3.0.

(a) Cases **(b)** Hospitalisations

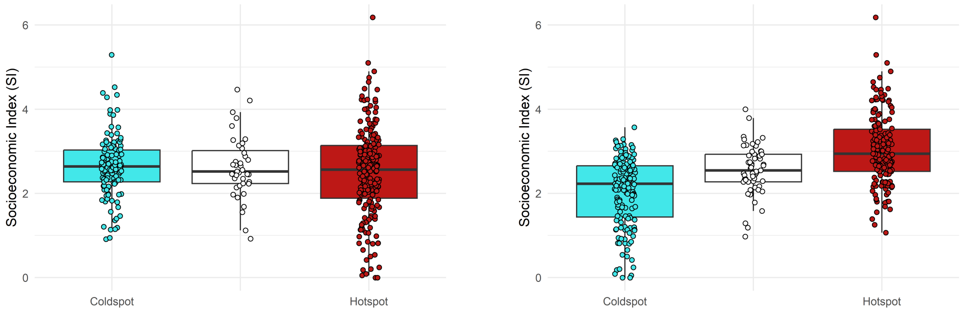

Supplementary Figure S3. Correlation matrix of the different socio-economic components. For all pairwise combinations of variables, a scatter plot of the pair of variables and the correlation between each of them is presented. Figure generated in R version 4.3.0.

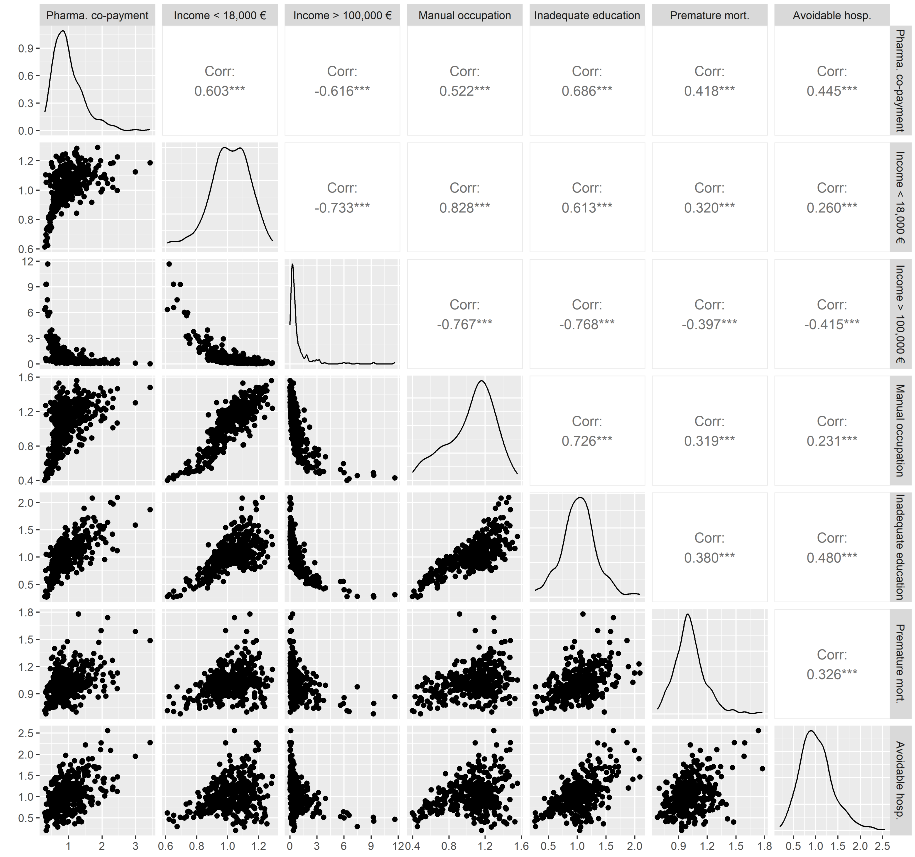

Supplementary Figure S4. Linearity of the relationship between socio-economic variables and COVID-19 cases. Scatter plot of the estimated spatial relative risk (RR) by the raw model for cases in function of each of the included socio-economic variables, together with the fitted Local Polynomial Regression Fitting curve. RR values are presented on a logarithmic scale. Areas that cannot be defined as either hotspots or coldspots are represented in white. Figures generated in R version 4.3.0.

(a) Socio-economic index

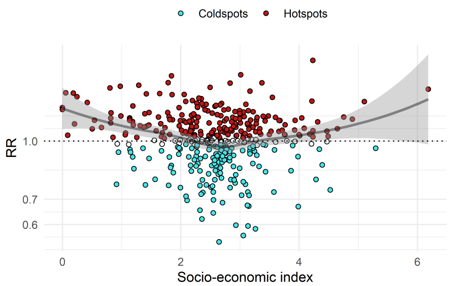

(b) Population exempted from pharmaceutical co-payment

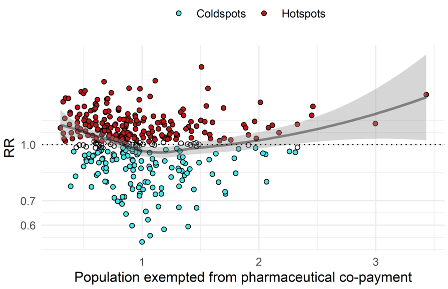

(c) Population with income < 18,000 €

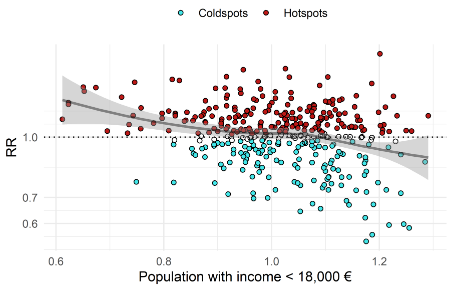

(d) Population with inadequate level of education

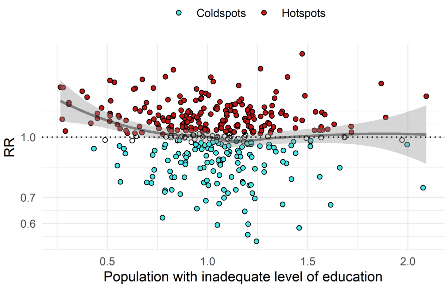

(e) Premature mortality

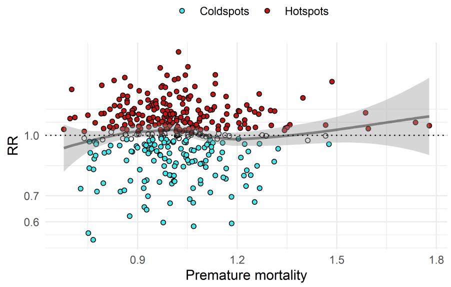

(f) Avoidable hospitalisations

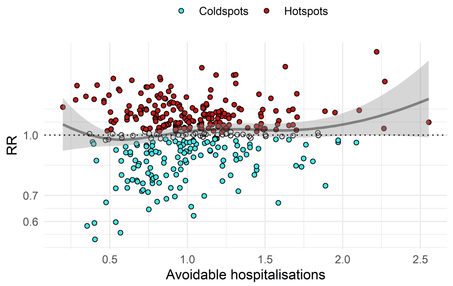

Supplementary Figure S5. Linearity of the relationship between socio-economic variables and COVID-19 hospitalisations. Scatter plot of the estimated spatial relative risk (RR) by the raw model for hospitalisations in function of each of the included socio-economic variables, together with the fitted Local Polynomial Regression Fitting curve. RR values are presented on a logarithmic scale. Areas that cannot be defined as either hotspots or coldspots are presented in white. Figures generated in R version 4.3.0.

(a) Socio-economic index

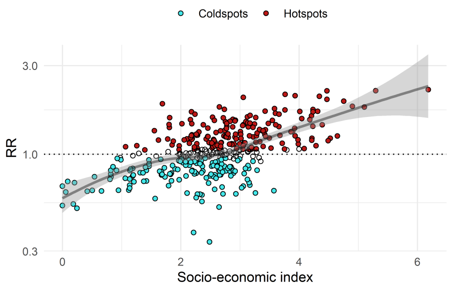

(b) Population exempted from pharmaceutical co-payment

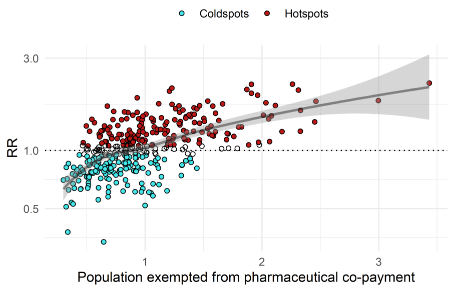

(c) Population with income < 18,000 €

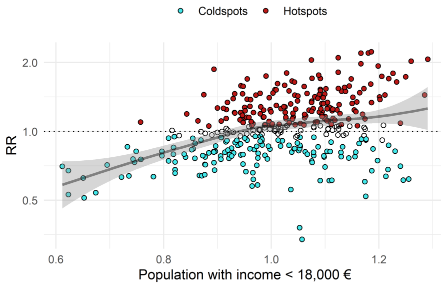

(d) Population with inadequate level of education

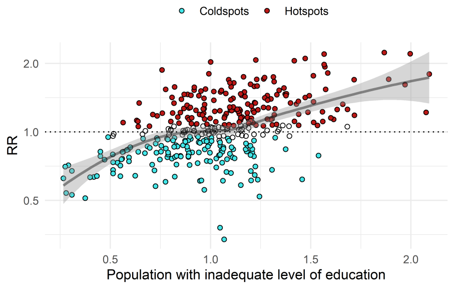

(e) Premature mortality

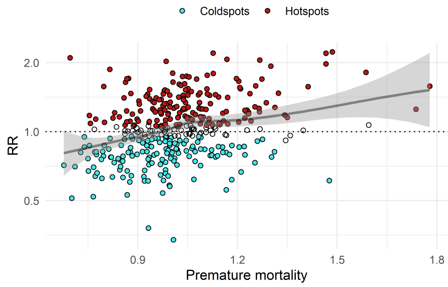

(f) Avoidable hospitalisations

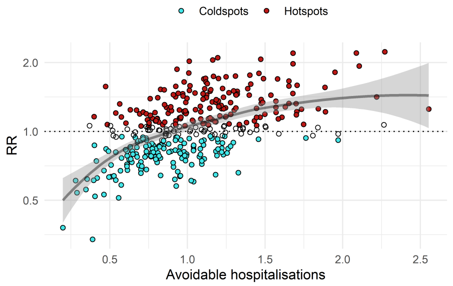

Supplementary Figure S6. Vaccination differences between hotspots/coldspots (waves 3-4). Boxplot of full vaccination percentages between hotspots and coldspots given by the spatial effect of the raw spatio-temporal model for hospitalisations over the waves 3-4. Areas that cannot be defined as either hotspots or coldspots are shown in white. Figure generated in R version 4.3.0.

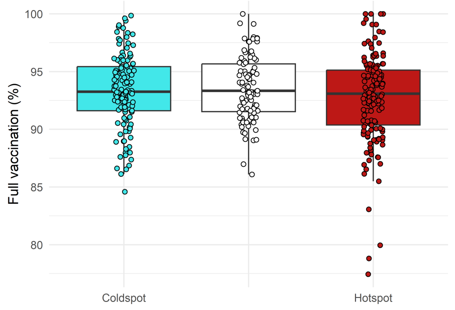

Supplementary Figure S7. Vaccination differences between hotspots/coldspots (wave 5). Boxplot of full vaccination percentages between hotspots and coldspots given by the spatial effect of the raw spatio-temporal model for cases and hospitalisations over the wave 5. Areas that cannot be defined as either hotspots or coldspots are shown in white. Figures generated in R version 4.3.0.

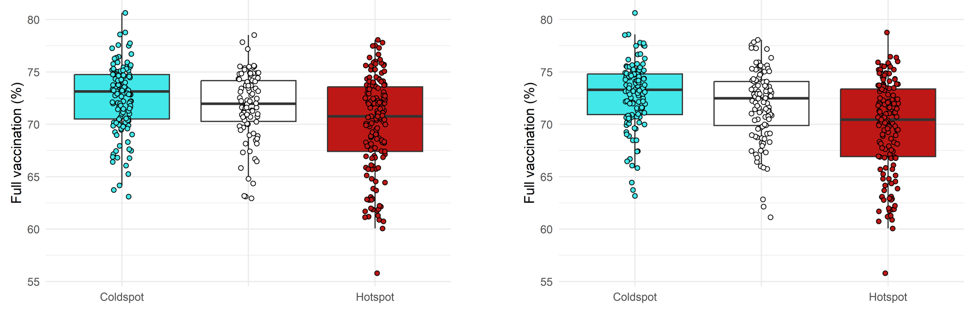

Supplementary Figure S8. Linearity of the relationship between vaccination and COVID-19 hospitalisations (waves 3-4). Scatter plot of the estimated spatial relative risk (RR) by the raw model for hospitalisations in function of the cumulative full vaccination percentage over the waves 3-4, together with the fitted Local Polynomial Regression Fitting curve. RR values are presented on a logarithmic scale. Areas that cannot be defined as either hotspots or coldspots are presented in white. Figure generated in R version 4.3.0.

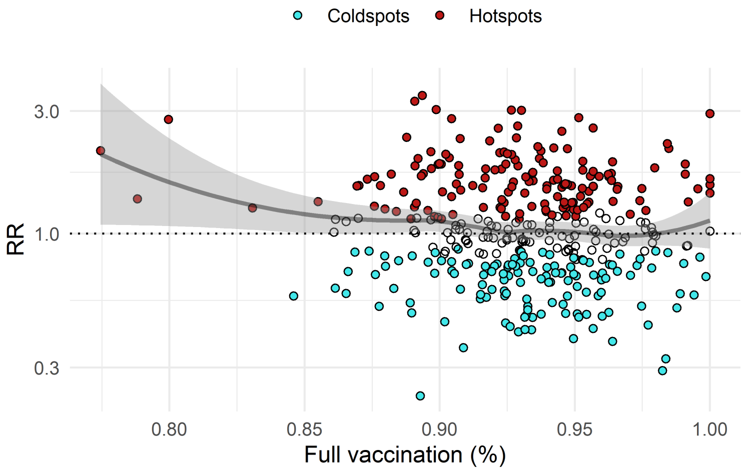

Supplementary Figure S9. Linearity of the relationship between vaccination and COVID-19 cases and hospitalisation (wave 5). Scatter plot of the estimated spatial relative risk (RR) by the raw model for cases and hospitalisations in function of the cumulative full vaccination percentage over the wave 5, together with the fitted Local Polynomial Regression Fitting curve. RR values are presented on a logarithmic scale. Areas that cannot be defined as either hotspots or coldspots are presented in white. Figures generated in R version 4.3.0.

(a) Cases

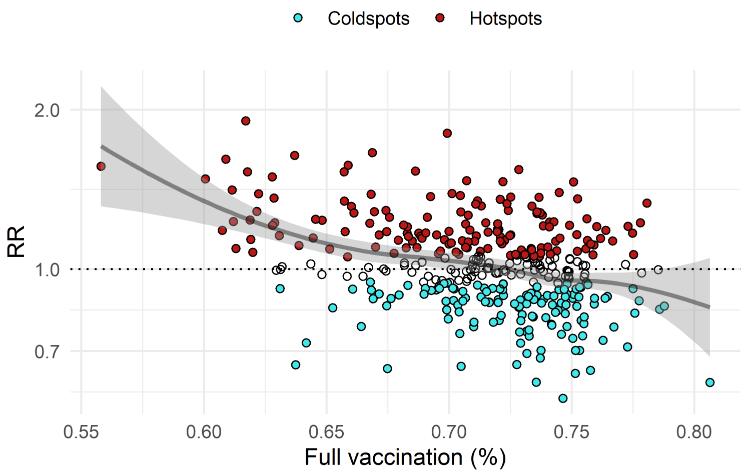

(b) Hospitalisations

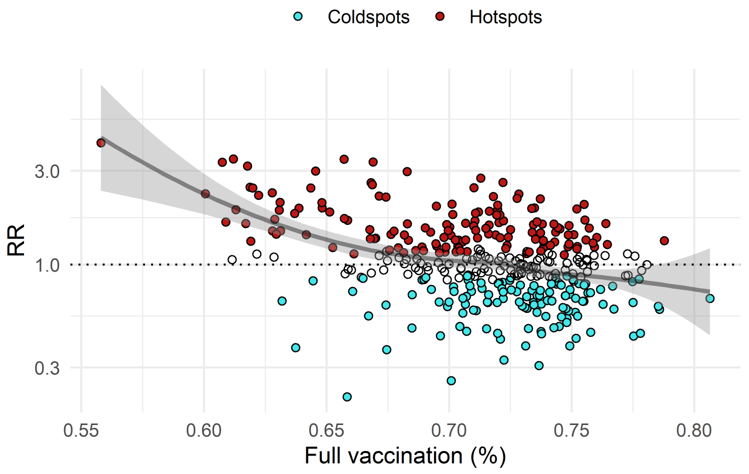

Supplement: Supplementary file 1 — Supplementary Information. [file 41598_2024_53527_MOESM1_ESM.pdf]
